# Harnessing Dendritic Cells for Tumor Antigen Presentation

**DOI:** 10.3390/cancers3022195

**Published:** 2011-04-26

**Authors:** Stefan Nierkens, Edith M. Janssen

**Affiliations:** 1 Department of Tumor Immunology, Nijmegen Centre for Molecular Life Sciences, Radboud University Nijmegen Medical Centre, Geert Grooteplein 28, Nijmegen 6525 GA, The Netherlands; E-Mail: s.nierkens@ncmls.ru.nl; 2 Division of Molecular Immunology, Cincinnati Children's Hospital Research Foundation, University of Cincinnati College of Medicine, 3333 Burnet Avenue, Cincinnati, OH 45229, USA

**Keywords:** dendritic cell, tumor antigen, antigen presentation, antigen processing

## Abstract

Dendritic cells (DC) are professional antigen presenting cells that are crucial for the induction of anti-tumor T cell responses. As a consequence, research has focused on the harnessing of DCs for therapeutic interventions. Although current strategies employing *ex vivo*-generated and tumor-antigen loaded DCs have been proven feasible, there are still many obstacles to overcome in order to improve clinical trial successes and offset the cost and complexity of customized cell therapy. This review focuses on one of these obstacles and a pivotal step for the priming of tumor-specific CD8^+^ and CD4^+^ T cells; the *in vitro* loading of DCs with tumor antigens.

## 1. Introduction

The overall goal of dendritic cell therapy is to generate *ex vivo* antigen-loaded DC that can stimulate robust and persistent CD8^+^ and CD4^+^ T cell responses to eliminate tumors and prevent metastases and recurrences. The rationale of the approach is that the efficiency of, and control over, the vaccination process provided by *ex vivo* manipulation of the DCs can generate an optimally potent antigen presenting cell (APC) and provide a superior method for stimulating anti-tumor immunity, compared to other immunization strategies.

The field of DC vaccines expanded exponentially after the establishment of techniques for *in vitro* generation of DC from mouse bone-marrow cells, human CD34+ hematopoietic progenitor cells, and human blood derived monocytes [1-7]. Currently there is a large body of literature involving animal models of tumor immunity in which DCs loaded with tumor-associated antigens (TAA) are able to induce protective immune responses. In addition, more than 300 trials have now utilized tumor-antigen loaded DCs as vaccines in humans—Yielding some clinical responses without any significant toxicity [8].

DCs are a phenotypically and functionally heterogeneous population of leukocytes with distinct functions. Over the years it has become clear that the conditions of DC generation and isolation greatly affect the potency of the DC vaccine. As elegant reviews on the effects of isolation and culture conditions on DC function have been recently published, these issues will not be addressed further in this review [9-11].

DC exist in 2 stages: Immature and mature. *In vivo*, immature DCs constantly monitor their environment, capture antigens, and display poor T cell-activating capacity due to their intermediate level of surface expression of MHC class I/II and low level of expression of costimulatory molecules (including CD80, CD86 and CD40). These immature DCs are not immunologically quiescent; they have been shown to induce T cell tolerance *in vivo* through the induction of T cell anergy, direct depletion of T cells, or by the generation of regulatory or suppressor cells that block the function of other effector T cells [12,13]. After antigen (Ag) uptake, DC migrate through the afferent lymphatics to draining lymph nodes. During this process DC start to mature, which is reflected by their decreased capacity for phagocytosis and increased expression of MHC and costimulatory molecules [14-16]. For full maturation and acquisition of T cell priming capacity DCs need to be “licensed”, which can occur by receiving pro-inflammatory signals in the form of CD4 T cell “help” through CD40-CD40L interaction [17-20], products that activate DCs via pathogen-associated molecular pattern (PAMP) receptors such as ligands for TLRs or NOD-LRR/CARD-helicases, and/or endogenous danger signals such as type I IFNs, HSPs, HMGB-1, or uric acid [21-24].

Adequate activation of T cells requires multiple signals from the DC to the T cell [25,26]. MHC-peptide recognition by the T cell receptor (TCR) on the T cell is crucial for initial activation, but will lead to anergy or non-responsiveness without appropriate additional costimulation provided by: (i) interaction between CD28 on the T cell and B7.1/B7.2 on the DC [27]; (ii) membrane-bound factors (including TRAF family members, CD40-CD40L, CD70-D27); and (iii) soluble factors such as cytokines and chemokines that support the survival of the T cells and, importantly, determine the phenotype of the activated T cells [27]. Although most DC populations become more immunostimulatory upon maturation, there is ample evidence that specific DC populations or specific maturation stimuli might still have the propensity to provide immunosuppressive or immunoregulatory responses.

Initial DC vaccine studies focused on the induction of cytolytic CD8^+^ T cell responses, but later research indicated that the activation of CD4^+^ T cells is required for optimal CD8^+^ T cell priming. CD8^+^ T cells primed in the absence of CD4^+^ T cell “help” yielded so-called “helpless” CD8^+^ T cells that exhibited diminished clonal expansion, cytokine production, and ability to lyse tumor cells [28-30]. More importantly, these “helpless” CD8^+^ T cells failed to develop into persistent memory cells and died after secondary encounter with antigen, due to TNF-related apoptosis inducing ligand (TRAIL)-mediated suicide [31]. In mouse models, secondary tumor challenge of “helpless” mice significantly reduced the tumor-specific CD8^+^ T cells and rendered the mice more susceptible to tumor grafting. Besides help in CD8^+^ T cell priming and maintenance, CD4^+^ T cells have been shown to recruit and activate various cell populations into the tumor environment, provide bystander mediated killing, and affect angiogenesis [32-37].

Although DC vaccine trials have been ongoing for almost 2 decades, clinical efficacy has been underwhelming. This is not surprising, as the use of DC therapy in tumor-bearing hosts places many demands on the DC vaccine.

The priming of tumor specific T cells is not a simple feat as tumors originate from “self” and self-reactive T cells are usually deleted by negative selection in the thymus. Self-reactive T cells that escape thymic selection display low affinity for the self antigen and are subject to peripheral tolerance. Moreover, many tumor-specific T cells have been rendered tolerant, anergic, or have been deleted from the repertoire by the immune suppressive machinations of the tumor and its microenviroment [38-42]. In addition, the *in vitro* manipulations needed to generate optimally potent DC have become a balancing act: Mature DC prime T cells, whereas immature DC can induce tolerance to the presented antigens [43-47]. However, maturation *in vitro* has also been shown to negatively affect DC survival and migration to T cell areas, and to significantly alter the *in vivo* production of cytokines that are relevant for T cell priming [48-51].

Given the large, pre-existing antigenic load and the immune suppressive mechanisms activated by tumors, optimal therapy should use properly activated DC that retain their migratory capacity and that are able to activate naïve T cells in the immune suppressive environment created by the tumor, as well as to reactivate and reinvigorate tumor-specific T cells that have been rendered tolerant or anergic by the tumor or its environment.

## 2. Tumor Antigens

Although most TAA originate from self, T cell responses to TAA are readily observed in patients and experimental animal tumor models. These antigens can be grouped into unique and shared antigens and originate from mutated proteins, abnormally expressed proteins and viral proteins.

Unique antigens encompass TAA that arise from mutations in normal gene products and have been suggested to result from the oncogenic process. Substitution mutations and chromosomal translocations provide most of the currently known TAAs while alternative ORFs, intron encoding products, and internal tandem repeats represent only a small percentage of the TAAs. As the mutations lead to the generation of neo-antigens, no pre-existing T cell tolerance should be present in the host, giving the opportunity for the induction of high affinity T cell responses during treatment. However, TAA are unique to the tumor of an individual patient or restricted to very few patients [52] and their use as immunotherapeutic targets may therefore be limited.

Shared antigens are present on many tumors and can be divided into tumor-specific antigens, differentiation antigens, and over-expressed antigens.

Shared tumor-specific Ag encompass antigens that are encoded by genes that are completely silent in most normal tissues but are activated in a variety of tumors and are generally related to the oncogenic process. This group also includes the “cancer-germline” antigens that are further only expressed in placental trophoblasts and testicular germ cells and viral antigens. The therapeutic potential of the shared tumor specific antigens is high as they are neo-antigens and no preexisting tolerance should exist.

Differentiation antigens are also expressed in the normal tissue of origin of the malignancy, while overexpressed antigens are expressed in a wide variety of normal tissues, but are higher expressed in tumors. Depending on expression pattern and level, T cell responses to these antigens will display a variety of affinities and levels of central and peripheral tolerance. The possibility of preexisting tolerance, and the risk of the development of autoimmune toxicity in healthy tissues, would make these more ubiquitously expressed Ag less favorable targets for therapy. However, the National Cancer Institute Pilot Project recently ranked 75 representative TAA based various criteria, including their (*i*) therapeutic function; (*ii*) immunogenicity; (*iii*) role in oncogenicity; (*vi*) specificity; (*v*) expression level and percent of antigen positive cells; (*i*) number of patients with antigen-positive cancers; (*vi*) number of antigenic epitopes; and (*vii*) cellular location of expression [53]. Interestingly, among the 10 highest ranking TAA, 5 could be characterized as “overexpressed antigens” (WT1, MUC1, Her-2/neu, p53, EGFRvIII) [53], suggesting that overexpressed TAA are still of considerable therapeutic interest.

## 3. Presentation in MHC I and II

The method of TAA loading is a critical step in DC therapy as it impacts: (*i*) the ability of the antigen to access both the MHC class I and II presentation pathways; (*ii*) MHC/peptide density; (*iii*) and MHC/peptide persistence on the DC [54-57]. Both CD4^+^ and CD8^+^ T cells require a minimum number of MHC/peptide-TCR interactions to become activated. Too few MHC/peptide-TCR interactions can result in T cell ignorance or tolerance, while too many interactions can lead to over-stimulation and subsequent induction of T cell anergy or T cell deletion.

### 3.1. MHCI Processing and Presentation

The majority of the antigens presented on MHCI are derived from endogenous cytosolic proteins that are ubiquitinated in the cytosol, fragmented in the proteasome and TAP-dependently transported to the endoplasmatic reticulum (ER). Under steady state conditions all somatic cells use ‘constitutive proteasomes’. Upon receiving inflammatory stimuli cells can replace the constitutive β1 (δ), β2 (MB1) and β5 (Z) subunits in the 20S core proteasome with three immunosubunits LMP2 (iβ1), LMP7 (iβ5) and MECL-1 (iβ2). The immunoproteasome displays reduced cleavage after acidic amino acids and increased cleavage after hydrophobic and basic residues [58], and is more likely to generate peptides with hydrophobic C-terminal residues [59]. Eventually eight to nine amino acid long peptides are loaded into newly synthesized MHCI molecules, and transported to the cell surface.

### 3.2. Cross-Presentation

Some cells have the unique ability to present antigens derived from the extracellular environment on MHCI via cross-presentation pathway, a process for which multiple non-exclusive mechanisms have been described [60-62]. In the *phagosome-to-cytosol pathway* the antigens are transferred from phagosomes into the cytosol, where they are cleaved by the general mechanisms used for endogenous proteins. Evidence for the latter was provided by findings that cross-presentation is dependent on the proteasome, TAP, and in some cases, ERAP-1 [63]. This pathway for cross-presentation benefits from a blockade of proteolyses in the phagosomes as this may increase the ‘leakage’ of intact antigens to the cytosol. The precise mechanism of antigen egress from phagosomes has not been deciphered yet, but a role for the retro-translocation activity of sec61 has been suggested [62]. In addition, loss of membrane stability by mediated by lipid bodies, which are thought to form the outer leaflet of the ER, have been implied in the escape of phagosomal content into the cytosol [64,65].

The antigens are subsequently degraded into peptides in the cytosol. Cross-presentation in most cases appears dependent on TAP. TAP was initially thought to be exclusively expressed in the ER and, therefore, antigen loading was thought to take place in the ER. However, TAP may also be recruited to the phagosomes, which gives rise to the possibility that peptides are retro-translocated to an ER-phagosome compartment before loading onto MHCI and transport to the cell membrane [66,67]. This pathway hence depends on the incomplete degradation of antigens in the phagosomes. Indeed, the ability to tightly regulate (increase) phagosomal pH in CD8α^+^ DCs (in comparison with for instance macrophages) has been suggested to be a decisive feature for the superior cross-presenting capacity of this DC subset.

In the *vacuolar pathway,* peptides are generated and loaded onto MHC-I immediately in the phagosome. Cross-presentation through this pathway is TAP-independent but inhibited by cysteine protease inhibitors, e.g., cathepsin S (catS) [62]. From experiments with TAP- or catS-deficient mice or DC, it can be concluded that the vacuolar pathway contributes less to cross-presentation than the phagosome-to-cytosol pathway.

### 3.3. MHCII Processing and Presentation

Peptides presented by MHCII typically originate from the extracellular space although the presentation of endogenous proteins-mostly from the cytosolic and nuclear compartments-has also been reported [68]. The α- and β-chain of MHC-II molecules are synthesized in the ER and associate with the invariant chain (Ii chain or CD74), which limits egress from the ER and prevents loading of self peptides. In DC most of these MHCIi complexes are directed to the cell surface, directly internalized, and either loaded with peptides or degraded in endocytic compartments. Most peptides are derived from cargo that has been internalized by endocytosis, a process for which APC have developed various mechanisms: Phagocytosis, macropinocytosis, clathrin-mediated endocytosis and non-clathrin/caveolae endocytosis [69]. What happens next is dictated by the antigenic content of the phagosome and by the presence of danger-associated molecular patterns, such as agonists for Toll-like receptors, which determine the recruitment of proteins and subsequent fusion to other vesicular bodies [70]. The final degradation of antigens occurs after the formation of the phagolysosome in which the acidification is tightly controlled by the activation of the APC. Peptides that are loaded in the MHCII groove are relatively large (10–20 AA), which fosters the suggestion that MHC molecules compete with lysosomal proteases for large peptide fragments. Once the MHC-peptide complex has been formed, the peptide is protected from further degradation.

## 4. Current Approaches

Several approaches have been used to generate TAA presenting DC, all with specific advantages and limitations.

### 4.1. Short Peptides

The early use of DC therapy employed the pulsing of DC with short (8–10 AA) peptides that represented CD8^+^ T cell epitopes of known TAA. Short peptides can bind directly to MHC and do not require antigen processing, allowing for highly controlled peptide loading of DC in all maturation stages. Many epitopes have been discovered [71] and the generation of clinical grade synthetic peptides is cheap and fast. Moreover, the use of synthetic peptides allows for modifications in the peptides. Terminal modifications in the form of acetylation or aminidation reduces proteolytic degradation, modification HLA-binding residues can increase the stability, while alterations of TCR interacting amino acids could increase TCR triggering. This “altered peptide” approach has been successfully used to design more immunogeneic peptides for epitopes in well known TAAs, including MART-A/Melan-A, gp100, Her-2/neu, NY-ESO-1 and CEA (CAP1-6D) [72].

However, there are several disadvantages of peptide therapy. The use of individual peptides requires the identification of specific epitopes for each HLA subtype. Whereas bioinformatic algorithms can predict HLA-binding TAA epitopes, these peptides are not necessarily expressed by the tumor and therefore may not function as rejection Ags [73]. In addition, it is likely that many relevant peptides would need to be optimized to prevent proteolytic degradation and reduction of the rapid turnover of surface MHC/peptide complexes. Although amino acid substitutions might overcome these problems, research has shown that such alterations do not necessarily translate in greater immunogenicity and that they carry the risk of reducing CTL responses [74]. Inclusion of CD4^+^ epitopes has been shown to increase the therapeutic potential of DC treatment in animal models as well as in clinical trials. However, whereas many CD8^+^ T cell epitopes are known, only a limited number of CD4^+^ T cell epitopes has been identified, and little work has been performed on their optimization.

### 4.2. Long Peptides and Proteins

The use of long peptides (>25 AA) and proteins could overcome many of the problems that occur in the short peptide loading. Long peptides and proteins contain both CD8 and CD4^+^ T cell epitopes that can be presented by a variety of MHC/HLA molecules obviating the need to identify patients' haplotypes. Long peptides can cover the natural sequence of a specific TAA or be designed to express specific epitopes from different TAAs. Importantly, research has shown that DC cross-presentation of proteins and extended peptides can last for days after antigen acquisition, due to storage of the antigens in a lysosome like depot compartment. This prolonged Ag-presentation would provide a greater window in which tumor-specific T cells can be recruited and interact with the DC.

Drawbacks of this approach include the need for TAA identification and the propensity of proteins to be targeted for MHCII presentation. Similar to short peptides, loading of proteins/long peptides fails to activate the DC, and therefore needs to be combined with a maturation stimulus. Given that DC predominantly use the immunoproteasome for Ag processing upon maturation, the peptide repertoire presented by the DC might change over time. Experimental studies have shown that HLA-A2 presentation of the well known TAA epitopes Melan-A_26-35_, gp100_209-217_, MAGE-A_161-169_, and MAGE-A3_168-176_ are heavily dependent on proteasome processing, while other HLA-2A restricted epitopes such as RAGE-A1_11-20_ can result from both proteasome and immunoproteasome processing [75-77].

### 4.3. Cell Lysates and Dying Cells

A great way to target all tumor antigens without the need for their identifications is the use of tumor cell lysates or dying tumor cells. Cell-associated antigens are efficiently cross-presented in MHCI, while also providing-albeit to a lesser degree–CD4 epitopes. Importantly, damaged and dying cells express many molecules that facilitate Ag uptake, enhance Ag processing and presentation, enhance DC maturation, or have an overall adjuvant effect in the priming of T cells. These so-called Danger Associated Molecular Patterns (DAMPS [78]) encompass plasma membrane components (including calreticulin, phosphatidylserine residues, and HSP70/90), cytosolic products (HSP70/90, uric acid, and high-mobility group box 1; HMGB1) and late stage degradation products such as DNA and RNA products [22,79-83]. One of the initial disadvantages of this approach, the need for a high volume of autologous tumor cells could be overcome using allogeneic tumor cells as TAA source.

Research has shown that the pathway of death determines the type of DAMPs that are released and thereby the immunostimulatory capacity of the corpse. Uptake of dying cells generated under normal homeostatic conditions inhibits maturation and pro-inflammatory cytokine production in the phagocytosing DC. In addition, the uptake of these apoptotic cells has been shown to induce immunoregulatory factors that dampen adaptive immune responses, including IL-10, transforming growth factor-β (TGF-β), and prostaglandin E_2_ [84-87]. This “tolerogenic” state has been shown to become entrenched in the DC program and, as a result, DC respond poorly or in an immune-suppressive fashion to subsequent immunostimulatory agents. Classic apoptosis has been suggested to provide fewer DAMPs than primary necrosis but is not necessarily always less immunogenic than necrosis. Late apoptotic events, as well as secondary necrosis have been shown to provide strong immunogenic stimuli as well. However, responses to dead and dying cells, or death associated DAMPs, may greatly vary among distinct DC populations.

Consequently, the identification of cell death pathways and cell death “stages” that enhance the immunogenicity of dying cells and prevent the induction of immunosuppressive mechanisms in DC populations has become a growing topic of research [88].

### 4.4. DC Tumor Fusion Approaches

An alternative approach to providing DC with TAA without the need for their identification is the fusion of whole tumor cells with syngeneic DC using polyethylene glycol, electrofusion or biological means [89-92]. The heterokaryote fusion product inherits the properties of both cells, and has been shown to produce, process and present TAA for days after the fusion. Similar to apoptotic cell loading, fusions can be made using allogeneic tumor cells as Ag presentation depends on the DC part of the fusion product. Tumor/DC fusion products have been extensively tested in animal models of solid and blood borne cancers, both in vaccination and therapeutic settings. In humans, over 20 Phase I/II clinical trials have been performed in patients with solid tumors including melanoma, glioma, renal cell carcinoma, breast cancer, ovarian cancer, gastric cancer [90]. Although the outcome in animal models is excellent, the clinical benefit in patients has been significantly less. Whereas animal studies use live fusion products, all clinical trials are performed with irradiated fusion products. It is likely that these products will be rapidly removed and targeted for cross-presentation. This idea is supported by the observation that the use of allogeneic DC for the fusion—Where CD8^+^ T cell priming would depend on the expression of MHC by the autologous tumor cell and appropriate MHC class II expression might be absent—Still shows immunogenicity and clinical benefit that is not significantly different from the use of autologous DC [90,93,94]. The use of live tumor/DC fusion products might result in better clinical efficacy, with the caveat that the fusion product might produce all of the immunosuppressive components of the tumor while interacting with the immune cells [95].

### 4.5. RNA Transfection

Introduction of TAA-encoding mRNA by electroporation, lipid mediated transfection or passive transfection is a simple, reproducible and effective method to generate TAA-presenting DCs. The mRNA can be designed to encode specific TAAs, or can be extracted from tumor cells to represent all TAAs and endow the DC with the capacity to generate antigenic peptides over an extended period of time. Clinical trials have shown the induction of T cell responses to a variety of TAAs, including PSA, TERT, CEA and OFA. In contrast to tumor fusion and apoptotic cells/lysate pulsing approaches that require significant amounts of (autologous) tumor cells for a sustained immunization protocol, only a small number of tumor cells is needed to isolate mRNA that can be amplified for subsequent uses. Moreover, incorporation or cotransduction of mRNA encoding molecules involved in DC trafficking, maturation, cytokine production/signaling and T cell activation can significantly enhance the induction of T cell responses [96-99]. In addition, the transfected mRNA can also function as stimulatory ligand for innate nucleotide sensors, leading DC maturation and upregulation of MHC and costimulatory molecules.

Whereas the lack of mRNA integration into the host genome is a tremendous regulatory advantage, it also results in limited stability and therefore a short lifespan of the mRNA in the DC. In addition, the mRNA-encoded antigens are preferentially targeted to the class I presentation pathway, leading to relatively poor CD4^+^ T cell responses. Altering the TAA by appending a leader sequence to the amino end and a lysosomal sorting signal to the carboxyl end has been shown to target more TAA to compartments where class II restricted peptides can be generated [100]. However, the effects of these alterations on CD4^+^ T cell stimulatory capacity are still modest [101,102].

### 4.6. DNA Transfer

TAA-encoding DNA can be transferred into DC using gene transfer vehicles such as cationic lipids, plasmid coated particles, electroporation and viral vectors that have been modified to eliminate the genes that encode replication factors [103,104]. Similar to RNA transfection, the TAA-encoding DNA can be combined with DNA for DC-associated molecules that provide positive signaling to T cells [105-107]. Viral transductions induce significantly higher levels of transgene expression than their non-viral counterparts and have a strong potential to activate cytosolic and endosomal innate sensors that result in activation and maturation signals in the DC [104]. TAA-expressing recombinant adenoviruses (AdV), pox viruses, retroviruses, and lentiviruses have been used in animal studies and clinical trials with various results [108]. Adenoviral vectors are widely used because of their high transduction efficiency and their consistent induction of both MHC class I and class II epitopes. However, *in vivo* studies show that proteins from the AdV backbone can dominate the immune response and might compete with the TAA-specific response [109,110]. Retroviruses also induce both CD8^+^ and CD4^+^ T-cell responses and have the advantage that the viral proteins are not expressed after integration of the transgene into the genome [111]. In spite of these advantages, retroviral transfection might be of limited clinical use as it only transduces dividing cells and can therefore only be used when DC are obtained from the more laborious CD34+ hematopoietic progenitor cell culture and not from blood derived monocytes [112,113]. Lentiviruses are a subclass of retrovirus and are able to transduce non-dividing cells [114]. Although levels of transgene expression are generally not as high as with other viral vectors, induction of TAA-specific CD4^+^ and CD8^+^ T cell responses is readily observed, and preclinical studies have been promising [115-117]. Lentiviral vectors are also of interest from the regulatory point of view: Although the site of vector integration is unpredictable and could hypothetically lead to disturbances in genes and promote the development of cancer, lentiviral vectors show low genotoxicity and low oncogenic potential compared to other viral vectors [118-121].

## 5. Conclusions

Over the years it has become clear that although TAA-expressing DC vaccines have potential, they have not lived up to their potential. DC population, preparation/isolation, maturation, and treatment variables as type of cancer, stage of cancer, treatment frequency, and route of administration have all shown to be of critical importance in the efficacy of DC therapy. Loading of TAA onto DC, just a small part of the DC vaccine process, clearly shows the complexity of the *in vitro* DC vaccine system. All methods of TAA loading have their own specific advantages and need to battle both method-specific and shared problems. Additional research on TAA-loading strategies that target both MHC class I and MHC class II is likely to improve the T cell priming capacity of the DC, but might still have limited impact on clinical outcomes without further optimization of the *in vitro* generation and maturation requirements of the DC.

An alternative for *in vitro* DC maturation is the rapid *in vivo* maturation of DC upon transfer. Depending on the DC population and the local pre-existing cytokine milieu, DC function can be significantly enhanced. Injections of immature DCs together with maturating stimuli, including type I IFNs or type I IFN-inducing PAMPs (especially nucleotide structures that engage TLR3, 7,or 9), are currently being tested [43,122-124].

The *in vivo* targeting of TAA to DC could overcome many of the problems that are associated with *in vitro* generation, loading and maturation of DC. Although this approach has tremendous potential, the identification of the DC population with the greatest therapeutic potential, the optimization of the TAA-configurations, and the targeting strategies in human patients is still in the early phase. Many of the *in vitro* TAA-loading approaches are not feasible (tumor fusion) or carry the risk of inducing tolerance (short peptides, dying cells). Other approaches that can be more easily tailored to directly target DC, such as viral vectors, face regulatory obstacles as well as the possibility of pre-existing immunity to vector-components and induced immunity to the vector that would interfere with repeat administrations [117,125].

Whether an *in vitro* or an *in vivo* TAA loading approach is used, DC still face the daunting task of priming or reactivating tumor-specific T cells in a hostile and immunosuppressive environment generated by the tumor and the host. Combination of DCs treatment with strategies that counteract the immune suppressive mechanisms of the tumor and host peripheral tolerance, or potentiate T cell function and survival should enhance the efficacy of DC therapy. Clinical studies interfering with regulatory T cells via depletion (ONTAK, IL-2/diphtheria toxin fusion protein, GITR, cyclophosphamide [126-129]) or by inhibiting their effector mechanisms (blocking IL-10, TGFβ) have been shown to add to a vaccine's efficacy. In addition, blocking of immune suppressive molecules expressed by the tumor or by DC (IL-10, TGFβ, PDL-1 [130,131]) are likely to improve T cell responses. Similarly, targeting molecules on tumor-specific T cells that are involved in the regulation of T cell survival and effector functions (e.g., IL-7, IL-15, activating antibodies to CD137, blocking CTLA-4 [132-135]) might translate into improved clinical outcomes.

It is likely there will never be a “one-size-fits-all” approach for DC tumor vaccines and that therapies will have to be tailored to the type and location of the tumor, the immune suppressive effects of the tumor, and the immune status of the patient. However, a multi-faceted approach that interferes with the immunesuppressive machinations of the tumor, mechanisms of host peripheral tolerance, and enhances T cell survival will likely provide the most advantageous stage for the protagonist role of the DC.
